# Triploidy in a fetus following amniocentesis referred for maternal serum screening test at second trimester

**DOI:** 10.4103/0971-6866.69371

**Published:** 2010

**Authors:** E. Bagherizadeh, M. Oveisi, Z. Hadipour, A. Saremi, Y. Shafaghati, F. Behjati

**Affiliations:** 1 Sarem Cell Research Center (SCRC), Sarem Women’s Hospital, Tehran, Iran; 2 Genetics Research Center, University of Social Welfare and Rehabilitation Sciences, Tehran, Iran

**Keywords:** Abnormal maternal serum screening test, prenatal diagnosis, triploidy

## Abstract

Amniocentesis was carried out at 17 weeks gestation in a 27-year-old woman, following an abnormal maternal serum screening (MSS) test. MSS test was carried out primarily to estimate the risk of trisomy for chromosome 21. The maternal serum markers used were alpha-fetoprotein (AFP), human chorionic gonadotrophin (hCG), and unconjugated estriol (uE3), together with maternal age. The fetus was identified as screen-positive for Edward’s syndrome (trisomy 18), with low uE3, normal AFP and hCG levels. The calculated risk for trisomy 18 was more than 1:50. To identify any possible chromosomal abnormality, cytogenetic investigation was carried out on the amniotic fluid sample. The fetus’s karyotype showed triploidy with 69, XXX chromosome complement in all the metaphase spreads obtained from three different cultures, using GTG banding technique. Upon termination of the fetus, gross abnormalities indicative of triploidy were present in the fetus.

## Introduction

Second trimester maternal serum alpha-fetoprotein (MS-AFP), human chorionic gonadotrophin (hCG), and unconjugated estriol (uE3) levels are evaluated in pregnancies to determine the risk of aneuploidy.[1,2]

Cases emerging as triploidy fall into two major groups with maternal serum screening (MSS) test. Pregnancies in the first group are those identifiable as screen-positive for both Down syndrome and an open neural tube defect (ONTD) with elevated MS-AFP, grossly elevated hCG, low/normal uE3, and probably elevated inhibin-A (INH-A). Pregnancies in the second group are identifiable as screen-positive for trisomy 18 with low/normal MS-AFP, and very low hCG, uE3, and INH-A.[3–7]

The most common clinical features of triploidy include intrauterine growth retardation, total syndactyly of third and fourth fingers, and central nervous system (CNS), heart, and renal defects. Hydatidiform mole, one of the characteristic features of pure triploidy, is found in more than 90% of cases.[8–12]

Pathologic examination of the umbilical cord of fetuses with chromosomal abnormalities indicated a single umbilical artery in some of the cases included.[13–15]

## Case Report

In this case, a woman at the age of 27 years and 16 weeks of gestation of her first pregnancy was referred for the evaluation of maternal serum markers to estimate the risk of aneuploidy.

Amniocentesis was performed at 17 weeks of pregnancy. Twenty milliliters of amniotic fluid sample was collected in sterile syringes. All procedures were performed transabdominally and under ultrasound guidance.

Amniocytes were removed from amniotic fluid by centrifugation. Three cultures were set up in sterile flasks (25 cm^3^) and Leighton tube. Culture work, slide preparation, and GTG banding were carried out following standard protocols. Microscopic analysis was carried out on 20 metaphase spreads using all the three cultures.

### Cytogenetic results

The karytotype was 69, XXX, consistent with a triploid fetus. Upon termination of the pregnancy, gross abnormalities were apparent in the 19-week fetus.

### Clinical examination

**Figure 1:**
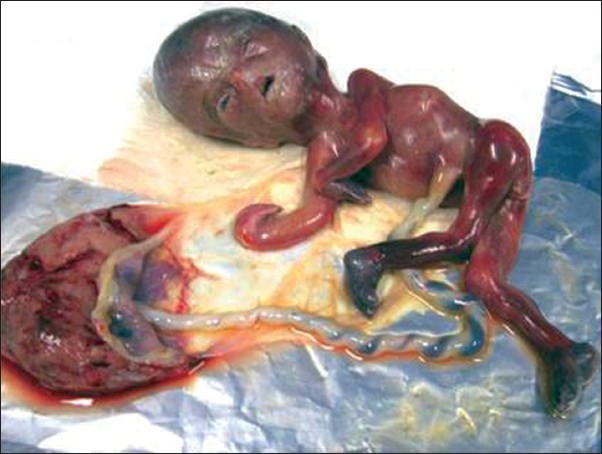
The terminated pregnancy showing different abnormalities

Gross examination of the 19-week fetus revealed a female fetus weighing 145 g, with external rotation within left forearm, internal rotation of left hip, and external rotation of left leg and umbilical cord containing two vessels; facial abnormalities included low-set ears and micrognathia [Figure 1].

Pathologic examination on fetus revealed agenesis of one lobe of the right lung and left kidney and the placenta contained one artery.

No pathologic findings were observed in lymphoreticular, hepatobiliary, cardiovascular systems, and gastrointestinal (GI) tract.

We present a fetus with an increased risk of trisomy 18 following an abnormal result of MSS test. The fetus was found to have a karyotype of 69, XXX, consistent with the second group of pregnancies with triploidy identifiable as screen-positive for trisomy 18. This is one of the few cases reported so far wherein due to abnormal MSS indicative of trisomy 18, the pregnancy was terminated as a case of triploidy.

The common clinical features of triploidy syndrome include severe intrauterine growth retardation, macrocephaly, total syndactyly of third and fourth fingers, and CNS, heart, and renal defects, dysplastic cranial bones, eye defects, cleft lip and/or palate, malformed ears, micrognathia, genital anomalies, asymmetric growth retardation, rarely spina bifida, hypotonicity, and atrophy of the cerebral cortex and corpus callosum.

In our case, the fetus had an abnormal appearance of placenta with two vessels and umbilical cord with one artery. There was agenesis in one lobe of the right lung and left kidney and facial anomalies with low-set ears and micrognathia. Other organs of lymphoreticular, hepatobiliary, cardiovascular systems were normal in pathologic examination.

Clinically, the most obvious finding was asymmetric skeletal growth of fetus which can be due to disproportionate prenatal growth deficiency which is consistent with other cases reported before.

An abnormal umbilical cord with a single umbilical artery in fetus’s placenta can be found phenotypically in cases of triploidy and other chromosomal abnormalities as reported previously in some fetuses with chromosomal abnormalities. A single umbilical artery (SUA) in the second trimester of pregnancy has a high association with trisomy 18, 13, 21 and other chromosomal defects; however it is not alone an indication for prenatal fetal karyotyping.

Our findings emphasize the use of maternal biochemical serum screening as a screening method for the detection of high risk pregnancies for chromosome abnormalities.
